# Human-supervised, large language model-based clinical decision support aligned to national newborn protocols in Kenya: a pragmatic, early-stage evaluation

**DOI:** 10.3389/fdgth.2026.1832634

**Published:** 2026-05-21

**Authors:** Teresiah Kuria, Gideon Kamau, Felisters Makokha, Protus Omondi, George Mbugua, David Kamau, Samuel Mbugua, Jesse Gitaka

**Affiliations:** 1 Centre for Research in Infectious Diseases, Mount Kenya University, Thika, Kenya; 2 Department of Research and Development, Labpoint Ltd, Thika, Kenya; 3 Department of Health and Sanitation, Ministry of Health, Bungoma, Kenya; 4 School of Nursing, United States International University in Africa, Nairobi, Kenya

**Keywords:** artificial intelligence, clinical decision support system, digital health, guideline adherence, human-supervised, Kenya, large language model, low-resource settings

## Abstract

**Introduction:**

Timely, protocol-adherent clinical decisions are crucial for reducing neonatal mortality in low-resource settings. Translating extensive national guidelines into bedside practice remains challenging.

**Objective:**

We developed and evaluated AIFYA, a human-supervised, large language model (LLM)-based clinical decision support system (CDSS) aligned with Kenya's national newborn care protocols.

**Methods:**

This prospective, mixed-methods, early-stage evaluation, guided by the DECIDE-AI framework, embedded AIFYA into routine workflows at two public health facilities (Level 5 and Level 4) in Bungoma County, Kenya, from September 2024 to June 2025. Primary outcomes were: (1) adoption, measured by cumulative neonatal cases managed; (2) training reach, assessed by credentialed healthcare workers (HCWs); and (3) guideline and citation concordance, evaluated through blinded review of 118 AI-generated recommendations by two neonatologists, with adjudication by a third. Secondary outcomes included protocol adherence and triage-to-decision time.

**Results:**

A total of 50 HCWs were trained, and 550 neonatal cases were managed over 10 months. Among surveyed HCWs (*n* = 33), 76% were female (mean age 32.1 years). Expert review found 75% of recommendations were correct and 15% partially correct, with strong inter-rater reliability (weighted Cohen's kappa 0.85; 95% CI 0.79–0.91) between reviewers. Citation accuracy was 96%. In 40 complex dosing scenarios, 75% of outputs were rated correct. The median triage-to-decision time was 23 min (IQR 18–31). Implementation was supported by an offline-first architecture and a facility-based coaching model, sustaining engagement despite staff turnover.

**Conclusion:**

A human-supervised, guideline-aligned AI CDSS can be feasibly implemented in routine neonatal care in low-resource settings, with high adoption and guideline concordance. Citation-linked recommendations enhance transparency and support clinical verification with most clinically appropriate recommendations. However, variability in complex scenarios highlights the need for ongoing refinement and strict clinical oversight. These findings support progression to control trials to evaluate clinical effectiveness and safety.

## Introduction

Each year, over two million newborns die within their first month of life, with the vast majority of these deaths occurring in low- and middle-income countries (LMICs) and from preventable causes (1). The persistence of this burden underscores a critical gap between established evidence-based guidelines and their consistent application in high-workload, low-resource clinical settings. In Kenya, the Ministry of Health has developed comprehensive and robust clinical practice guidelines the Comprehensive Newborn Care Protocols (2) and Basic Paediatric Protocols (3) to standardize care. However, the cognitive burden of navigating these extensive documents during urgent clinical events remains a significant barrier to implementation.

Artificial intelligence (AI), particularly large language models (LLMs), offers a transformative opportunity to bridge this evidence-to-practice gap by delivering synthesized, context-aware decision support at the point of care (4). Yet, the deployment of AI in health, especially “black-box” systems, raises legitimate concerns regarding safety, accountability, algorithmic bias, and the potential to undermine clinical autonomy (5, 6). Global consensus, articulated through reporting guidelines like DECIDE-AI, CONSORT-AI, and STARD-AI, emphasizes the need for a staged, transparent, and rigorous evaluation pathway for AI interventions, beginning with early-stage studies focused on feasibility, accuracy, and workflow integration (7–9).

To address these needs, we developed AIFYA, an LLM-powered CDSS co-designed with frontline clinicians and meticulously aligned with Kenyan national guidelines. AIFYA's core design principle is transparency: every recommendation is accompanied by a precise citation to the source protocol, mandating a human-in-the-loop workflow where the clinician verifies the guidance and retains final authority.

This paper reports the findings of a pragmatic, early-stage evaluation of AIFYA's implementation in a real-world county health system. Our objectives were to: (1) quantify adoption and training reach; (2) rigorously evaluate the guideline and citation concordance of AI outputs through independent, de-identified expert review; (3) characterize process performance and key implementation determinants; and (4) establish a robust evidence base to inform the design of future, definitive clinical trials.

## Materials and methods

### Study design and governance

We conducted a prospective, mixed-methods implementation study from September 1, 2024, to June 30, 2025. The study design and reporting adhere to the DECIDE-AI (Reporting guideline for the early-stage clinical evaluation of AI decision support) framework (8). Ethical approval was obtained from the Mount Kenya University Institutional Ethics Review Committee (MKU/IERC/3425), and administrative approval was granted by the Bungoma County Health Management Team (CHMT).

### Setting and participants

The study was conducted out in three public health facilities in Bungoma County, a rural area in Western Kenya with a high burden of neonatal morbidity and mortality. The sites comprised one Level 5 facility (Bungoma County Referral Hospital) and two Level 4 sub-county hospitals (Webuye and Kimilili). Participants were healthcare workers (HCWs); including nurses, clinical officers, and medical officers, who were responsible for neonatal care in the participating facilities. All HCWs who provided care in the newborn units during the study period were eligible for training and participation. Patient data used for system evaluation and process outcomes were included only if informed consent for data use had been secured from the newborn's parents.

### The AIFYA intervention

AIFYA is a tablet-based clinical decision support system (CDSS) that integrates a fine-tuned generative large language model (LLM) based on the GPT-4 architecture with a structured knowledge base derived from the 2022 Kenya Consolidated Newborn Clinical Protocols (CNCP) and Basic Paediatric Protocols. Clinicians input both structured and unstructured patient data, including vital signs, gestational age, and clinical observations. The system then generates a prioritized checklist of recommendations for assessment, diagnosis, and management, organized by clinical pathway (e.g., neonatal sepsis, jaundice, prematurity).

### Key features

The AIFYA platform incorporates several key design features to ensure safe, transparent, and context-appropriate clinical decision support.

First, it employs a mandatory human-in-the-loop workflow in which clinicians must review and either acknowledge, modify, or reject each AI-generated recommendation before proceeding, with all interactions and overrides automatically logged for audit and supervisory purposes.

Second, every recommendation includes a direct hyperlink to the exact page and section of the Ministry of Health newborn care protocols, enabling immediate verification and enhancing citation correctness and transparency.

Third, the system is built on an offline-first architecture tailored for low-connectivity environments: encounter data are stored locally on the device and automatically synchronized with a secure cloud server once internet access becomes available.

Finally, AIFYA incorporates embedded clinical safety guardrails, including weight-based dose calculators with range checks and automated alerts for contraindications and critical “red-flag” signs requiring urgent escalation.

### Training and implementation strategy

Healthcare workers (HCWs) participated in a two-day, hands-on training workshop covering both AIFYA system use and a refresher on the Kenya Consolidated Newborn Care Protocols (CNCP). Competency was assessed using a post-training test, with a score of ≥80% required for credentialing as an AIFYA user.

To support real-world implementation, the training emphasized not only system navigation but also integration of AI-supported recommendations into routine clinical workflows, including triage, assessment, and decision-making processes.

To mitigate the effects of staff turnover and sustain adoption, a facility-based coaching model was implemented, wherein trained peer champions provided ongoing mentorship and onboarding support for new staff. This approach facilitated continuous engagement with the system and supported its incorporation into day-to-day clinical practice.

### Outcomes and data collection

The primary outcomes were adoption, training reach, and guideline concordance of AI-generated recommendations. Guideline concordance was assessed through independent expert review of AIFYA-generated outputs against national newborn care protocols.

Secondary outcomes captured implementation and process-level performance, including protocol adherence over time, triage-to-decision time as a measure of workflow efficiency, and healthcare worker (HCW) perceptions of system usability and acceptability.

Data sources comprised AIFYA system logs (capturing user interactions, timestamps, and cases managed), training attendance registers, credentialing exam results, and structured expert review forms. HCW perceptions of AI and system usability were collected through an anonymized post-implementation survey (*n* = 33).

### Expert review process

A purposive sampling strategy was used to select cases representing common neonatal conditions (sepsis, jaundice, prematurity, hypothermia, and birth asphyxia), as well as variation in clinical complexity, including routine and complex dosing scenarios. Cases were selected to ensure completeness of clinical inputs and corresponding AI-generated outputs, while providing representation across the study period and participating facilities. Each scenario corresponding AI-generated recommendations was independently reviewed by two board-certified neonatologists. Reviewers conducted their assessments independently without access to each other's ratings, and discrepancies were resolved by a third reviewer. Reviewers were aware that the recommendations were AI-generated. The concordance evaluation focused on AI-generated recommendations produced by the AIFYA system. Expert reviewers assessed these outputs for correctness and alignment with national newborn care protocols. This assessment did not include evaluation of whether individual recommendations were accepted or implemented by healthcare workers in clinical practice.

### Recommendations were rated on two dimensions

Recommendation Correctness: Classified as *Correct* (fully aligned with standard of care), *Partially Correct* (clinically safe but incomplete or suboptimal), or *Incorrect* (potentially unsafe or contradicting guidelines).

Citation Correctness: Rated as *Correct* or *Incorrect* depending on whether the cited reference accurately supported the recommendation.

Discrepancies between reviewers were resolved by a third senior neonatologist.

### Statistical analysis

Descriptive statistics were computed for adoption, training, and HCW demographics. For the primary concordance outcome, percentage agreement was reported, and inter-rater reliability was estimated using linearly weighted Cohen's kappa to account for the ordinal scale of agreement between reviewers. Ninety-five percent confidence intervals (CIs) were generated via non-parametric bootstrapping with 1,000 resamples.

Qualitative data, derived from Key Informant Interviews (KIIs) and expert review free-text comments, underwent transcription and subsequent translation as required. A thematic framework approach governed the analysis. Specifically, two independent researchers coded the transcripts and inductively developed a comprehensive analytical codebook. Discrepancies in coding were resolved through consensus involving a third senior researcher. Dominant themes were systematically identified, revolving around usability, clinician confidence, workflow integration, and areas of clinical disagreement. Each thematic category is substantiated by the selection of illustrative quotations.

To assess changes in protocol adherence over time, we used a logistic regression model including fixed effects for facility to account for clustering. Sensitivity analyses using random intercept models yielded similar results. All statistical analyses were conducted using R statistical software (version 4.5.2; R Foundation for Statistical Computing).

## Results

### Adoption, reach, and healthcare worker characteristics

Between September 2024 and June 2025, a total of 532 neonatal cases were managed using the AIFYA clinical decision support system (CDSS) across the four participating facilities. The newborn and maternal characteristics are summarized in Table 1. Overall, 50 healthcare workers (HCWs) were trained and credentialed to use the AIFYA platform, and 33 of these participated in the endline survey. The mean age of respondents was 32.1 years (95% CI: 29.4–34.8). Most participants were female (76%). Nurses constituted the largest professional group (33%), followed by clinical officers (27%), other allied health staff (28%), and medical officers (12%). Nearly half of the respondents (48.5%) had prior digital health training, while none reported previous experience with artificial intelligence (AI) or CDSS tools (Table 2).

**Table 1:** Newborn and maternal characteristics across hospitals.

Characteristics	Total (*N* = 532)	Bungoma DH (*N* = 452)	Webuye CH (*N* = 58)	Kimilili S-BH (*N* = 22)	*p*-value
Age (days), mean ± SD	2.20 ± 3.56	2.22 ± 3.61	2.14 ± 3.71	1.82 ± 1.62	0.410
Birth weight (g), mean ± SD	2,346.5 ± 899.3	2,330.9 ± 914.0	2,306.2 ± 776.2	2,774.5 ± 819.0	<0.001
Gender, *n* (%)					0.563
Female	222 (41.7)	185 (40.9)	28 (48.3)	9 (40.9)	
Male	310 (58.3)	267 (59.1)	30 (51.7)	13 (59.1)	
Mother's age (years), mean ± SD	25.0 ± 5.9	25.1 ± 6.0	25.2 ± 5.5	23.4 ± 3.5	0.433

Values are expressed as mean ± standard deviation (SD) for continuous variables and as number (percentage) for categorical variables. *p*-values were obtained using the Kruskal–Wallis test for continuous variables (due to unequal group sizes and potential non-normality) and chi-square or Fisher's exact test for categorical variables, as appropriate.

**Table 2:** Characteristics of participating healthcare workers.

Characteristic	*n* (%) or Mean (95% CI)
Age (years)	32.1 (29.4–34.8)
Sex	
Female	25 (76%)
Male	8 (24%)
Professional role	
Nurse	11 (33%)
Clinical Officer	9 (27%)
Medical Officer	4 (12%)
Other allied staff	9 (28%)
Prior digital health training	16 (48.5%)
Previous AI/CDSS experience	0 (0%)

### Guideline and citation concordance

**Figure 1:**
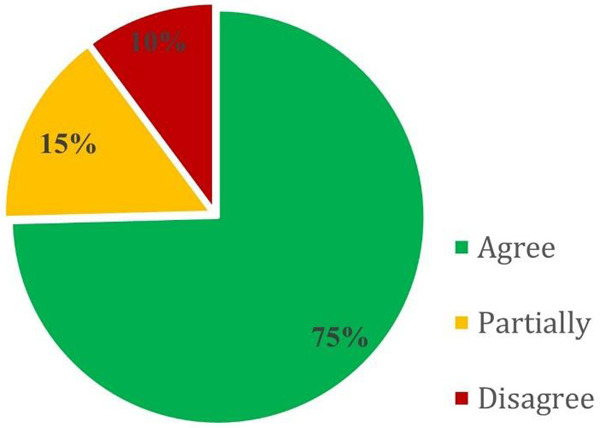
Distribution of AIFYA-generated recommendation ratings. Ratings were categorized as *Agree*, *Partially Agree*, or *Disagree* across 118 selected expert-evaluated clinical scenarios.

Independent expert evaluation was conducted to assess the correctness of AIFYA-generated neonatal management recommendations against the Kenyan national newborn care protocols. Across 118 clinical scenarios, 75% of the recommendations were rated as correct, 15% as partially correct, and 10% as incorrect (Figure 1). The overall agreement between AIFYA outputs and expert assessments was strong, with a linearly weighted Cohen's kappa of 0.85 (95% CI: 0.79–0.91). Citation accuracy was also high, with 96% of references correctly cited.

In a focused analysis of 40 complex dosing and fluid management scenarios, experts rated 75% (*n* = 30) as correct, 15% (*n* = 6) as partially correct, and 10% (*n* = 4) as incorrect (Table 3). Most discrepancies arose from weight-based adjustments for extremely preterm infants, and these insights were subsequently used to refine the system's dosing algorithms and safety guardrails.

**Table 3:** Expert review of AIFYA-generated recommendations: agreement and citation accuracy.

Domain	Correct *n* (%)	Partially Correct *n* (%)	Incorrect *n* (%)	Weighted *κ* (95% CI)
Overall (*n* = 118 scenarios)	88 (75%)	18 (15%)	12 (10%)	0.85 (0.79–0.91)
Citation correctness	113 (96%)	–	5 (4%)	–
Focused dosing scenarios (*n* = 40)	30 (75%)	6 (15%)	4 (10%)	–

Recommendation correctness was classified as correct, partially correct, or incorrect based on expert evaluation of AI-generated recommendations against national newborn care protocols. Citation accuracy reflects whether the cited guideline appropriately supported the recommendation. Inter-rater reliability (weighted Cohen's kappa) reflects agreement between the two independent expert reviewers.

### Process performance and implementation fidelity

Protocol adherence, assessed using a standardized checklist of essential newborn care actions, showed a statistically significant improvement over the study period. The odds of adherence increased by 5% per month (OR = 1.05; 95% CI: 1.02–1.08; *p* = 0.004). However, given the observational design and absence of a control group, this trend should be interpreted descriptively and cannot be attributed to the AI CDSS. The median time from patient triage to the first documented clinical decision was 23 min (IQR 18–31), and this performance metric remained stable throughout the evaluation period.

The system's offline-first architecture ensured continuity of service, with 35% of all clinical sessions conducted fully offline due to intermittent internet connectivity. User satisfaction was high, with 92% of healthcare workers (HCWs) agreeing or strongly agreeing that AIFYA was a useful tool in their daily clinical work (Table 4).

**Table 4:** Implementation and process performance outcomes.

Metric	Result (95% CI or IQR)	Comment
Total neonatal cases managed via AIFYA	532	Across both study sites
HCWs trained and credentialed	50	Two-day training program
Triage-to-decision time (minutes)	23 (IQR 18–31)	Stable throughout study
Sessions completed offline	35%	Offline-first functionality
Change in guideline adherence over time (aOR per month)	1.05 (1.02–1.08), *p* = 0.004	Logistic regression model adjusted by facility
User satisfaction (“AIFYA is useful”)	92% agree or strongly agree	Based on 5-point Likert survey

Process and implementation metrics summarizing adoption, system performance, and user experience during the 10-month evaluation. aOR = adjusted odds ratio; CI = confidence interval. Logistic regression models included fixed effects for facility.

### User perceptions of AI and human oversight

Survey data revealed strong support for a human-governed AI model as summarized in Table 5. A majority of HCWs (79%) rated human oversight of AI-assisted recommendations as “extremely important” or “very important.” The most frequently cited concern regarding AI integration was the potential for clinical over-dependence on technology (51.5%), followed by concerns about the accuracy of AI-generated recommendations (30.3%) (Table 5).

**Table 5:** Healthcare worker perceptions of AI use and human oversight in clinical decision-making.

Domain	Response category	*n* (%)	Comment
Perceived importance of human oversight	Extremely important	11 (33.3)	Majority emphasize strong human role
	Very important	15 (45.5)	
	Moderately important	5 (15.2)	
	Slightly/Not important	2 (6.0)	
Primary concerns about AI integration	Risk of clinical over-dependence	17 (51.5)	Most cited concern
	Accuracy of AI-generated recommendations	10 (30.3)	
	Data privacy and security	4 (12.1)	
	Other concerns	2 (6.1)	e.g., workflow disruption
Perceived usefulness of AIFYA	Agree/Strongly agree	30 (92.0)	Indicates high user satisfaction
	Neutral/Disagree	3 (8.0)	

## Discussion

In this pragmatic, early-stage evaluation, we found that a human-supervised, large language model (LLM)–based clinical decision support system (CDSS), carefully aligned with national newborn care guidelines, achieved high adoption, strong clinical concordance, and reliable technical performance when deployed in routine neonatal units in rural Kenya. Together, these findings highlight the feasibility, safety, and acceptability of integrating generative AI tools into frontline clinical workflows in low-resource settings.

First, adoption and reach were substantial, with 550 neonatal cases managed using AIFYA across four facilities and 50 healthcare workers (HCWs) trained and credentialed. The high utilization and satisfaction rates (92% of users agreeing that AIFYA was useful) underscore strong user engagement and confidence in the system. This success was facilitated by a socio-technical design that combined robust offline-first functionality essential in settings with intermittent connectivity with a human-led coaching model. Our findings are consistent with growing literature demonstrating that responsible AI integration, strong human oversight, and context-appropriate design can strengthen clinical practice in resource-constrained environments (10, 11).

Second, clinical and technical performance was consistently strong. Independent expert evaluation revealed 75% correct and 15% partially correct recommendations, with a high overall agreement (*κ* = 0.85) relative to national newborn protocols. Citation accuracy reached 96%, introducing a novel and transparent measure of “explainable correctness” that enables clinical supervision and fosters user trust. This level of concordance is consistent with emerging evidence showing that well-supervised AI systems can reliably align with established clinical guidelines (12, 13). However, the observed difference between high citation accuracy and slightly lower clinical correctness highlights an important distinction between guideline traceability and contextual appropriateness. While recommendations were frequently supported by appropriate guideline references, this did not always translate into clinically optimal decisions for individual patients. This discrepancy was most evident in complex scenarios, particularly weight-based dosing in extremely preterm infants, where nuanced clinical judgment is required.

Importantly, the concordance analysis in this study reflects the technical validity of AI-generated recommendations, rather than the final clinical decisions made by healthcare workers. While high agreement with national guidelines is encouraging, it does not necessarily indicate that recommendations were consistently accepted or implemented in practice. This distinction is critical when interpreting the findings within the context of real-world clinical care.

Third, process performance and implementation fidelity demonstrated meaningful improvement in protocol adherence over time. An increase in protocol adherence was observed over time. However, this finding should be interpreted cautiously. Given the observational design, absence of a control group, and potential influences such as Hawthorne effects and secular trends, it is not possible to attribute this change to the AI CDSS. Rather, this trend may reflect a combination of factors, including training effects, increased familiarity with clinical protocols, or broader temporal variation. The median triage-to-decision time (23 min) remained stable throughout the study, indicating that AIFYA integrated smoothly into clinical workflows without introducing delays. Sustaining digital health tools within large and complex health systems requires careful attention to adoption and implementation factors, as documented in previous studies (14–16). Therefore, future controlled studies with baseline comparisons will be required to determine whether AIFYA-supported decision-making contributes to improvements in protocol adherence.

Finally, user perceptions of AI and human oversight highlighted both enthusiasm and caution among frontline clinicians. The majority emphasized that human oversight of AI-assisted recommendations is “extremely” or “very” important, reflecting a nuanced understanding of AI's role as a decision-support not decision-making tool. The most frequently cited concerns, included clinical over-dependence and potential inaccuracies, reinforcing the importance of maintaining a mandatory human-in-the-loop framework. Such concerns mirror well-documented risks of automation bias and error propagation in digital decision-support systems, particularly in resource-constrained settings (10, 17, 18).

Collectively, these findings provide one of the first real-world examples of an ethically grounded, explainable, and context-adapted AI system deployed in a low-resource clinical setting. By pairing technical innovation with human supervision and local guideline alignment, AIFYA demonstrates a feasible and scalable approach that may integrate AI-based decision support into routine care.

While the system showed strong adoption, high user acceptability, and encouraging levels of guideline concordance, these findings primarily reflect technical performance and workflow integration. Further evaluation is required to determine the extent to which AI-generated recommendations influence clinical decision-making and improve patient outcomes in real-world settings.

### Strengths and limitations

The primary strength of this study lies in its pragmatic design, which evaluated the system under authentic clinical conditions in rural neonatal units. The study's adherence to the DECIDE-AI reporting framework enhances methodological transparency and reproducibility. Additional strengths include the rigorous, independent, multi-expert review of AI-generated recommendations, providing a structured assessment of guideline concordance, and the introduction of “citation correctness” as a novel performance metric for explainability and accountability in LLM-based clinical decision support systems.

The study also incorporated process-level and implementation outcomes, including protocol adherence over time, triage-to-decision time, and healthcare worker perceptions, offering complementary insights into workflow integration and system acceptability in routine care settings.

Several limitations should be acknowledged. First, as an early-stage evaluation, the study was not powered to assess downstream clinical outcomes such as mortality, morbidity, or length of stay. Second, the concordance assessment focused on AI-generated recommendations rather than final clinical decisions and thus does not capture clinician adherence to recommendations or real-world decision-making at the patient level. Observed improvements in protocol adherence over time cannot be causally attributed to the intervention due to the absence of a control group and potential confounding factors, including temporal trends and observational bias. Although cases spanned key neonatal conditions, the findings may not be fully generalizable to all neonatal presentations or other healthcare contexts. Additionally, while the offline-first architecture was effective, long-term sustainability and integration within national digital health systems will require further evaluation at scale.

## Conclusion

A human-supervised AI clinical decision support system aligned with national newborn care protocols can be feasibly implemented within routine, low-resource neonatal care settings. The system demonstrated strong adoption, high user acceptability, and encouraging levels of guideline concordance. However, the slight variability in recommendation correctness, particularly in complex dosing scenarios, highlights the need for continued refinement and careful clinical oversight. These findings support progression to controlled, multi-site trials to evaluate the system's impact on clinical decision-making, patient outcomes, and safety.

## Data Availability

The datasets presented in this study can be found in online repositories. The names of the repository/repositories and accession number(s) can be found in the article/Supplementary Material.
