# Supplementary material for: Human-supervised, large language model-based clinical decision support aligned to national newborn protocols in Kenya: a pragmatic, early-stage evaluation
Source: Front Digit Health. 2026 May 21;8:1832634. doi: 10.3389/fdgth.2026.1832634 (PMC13233492; doi:10.3389/fdgth.2026.1832634)
Supplement: Supplementary file 1 [file Supplementaryfile1.docx]

**Supplementary material: Human-supervised, large language model-based clinical decision support aligned to national newborn protocols in Kenya: a pragmatic, early-stage evaluation**

**1. Objectives of the Expert Review**

This evaluation was conducted by blinded, board-certified neonatologists to quantify the performance and reliability of the AIFYA system against the Kenya National Newborn Clinical Protocols (CNCP). The specific objectives were:

- Recommendation Correctness: To precisely measure the degree of agreement between AI-generated recommendations and established guideline recommendations (accuracy).
- Citation Accuracy: To measure the correctness and precision of the system's citation output (document and specific page reference).
- Error Pattern Identification: To identify and characterize patterns of systematic errors, such as consistent miscitations to particular guideline sections or incorrect clinical interpretations.
- Transparency Assessment: To assess the system's transparency by comparing the cited guideline content against the actual content used to support the recommendation.

**2. Analysis Plan and Key Accuracy Metrics**

The following metrics were used to determine the core accuracy of the AIFYA system's outputs:

- Recommendation Accuracy: Defined as the percentage of AI-generated recommendations that were classified by expert reviewers as fully consistent with the established guideline recommendations.
- Citation Accuracy: Defined as the percentage of citations that correctly matched both the referenced document and the specific page number of the relevant national protocol.

**3. Evaluation Framework and Statistical Metrics**

The statistical evaluation of inter-rater reliability and final agreement utilized a robust framework:

Inter-Rater Reliability Metrics:

- Cohen’s Kappa ($\kappa$): Both unweighted (for nominal data) and linearly weighted (for ordinal recommendation correctness data) were computed to assess agreement beyond chance.
- Observed Agreement ($P_o$): The raw percentage of cases where the reviewers agreed.
- Expected Agreement ($P_e$): The probability of agreement occurring by chance.
- Confidence Intervals:
- 95% Confidence Intervals (CIs): Generated for all primary agreement metrics to indicate the precision of the estimate.

4. Sample Cases Review

A set of sample cases illustrating the Agreement Metrics for AI vs. National Guideline evaluation process are provided below. The figures below demonstrate the comparison of clinician inputs, AI outputs, and cited pages from the National guidelines for newborn care.


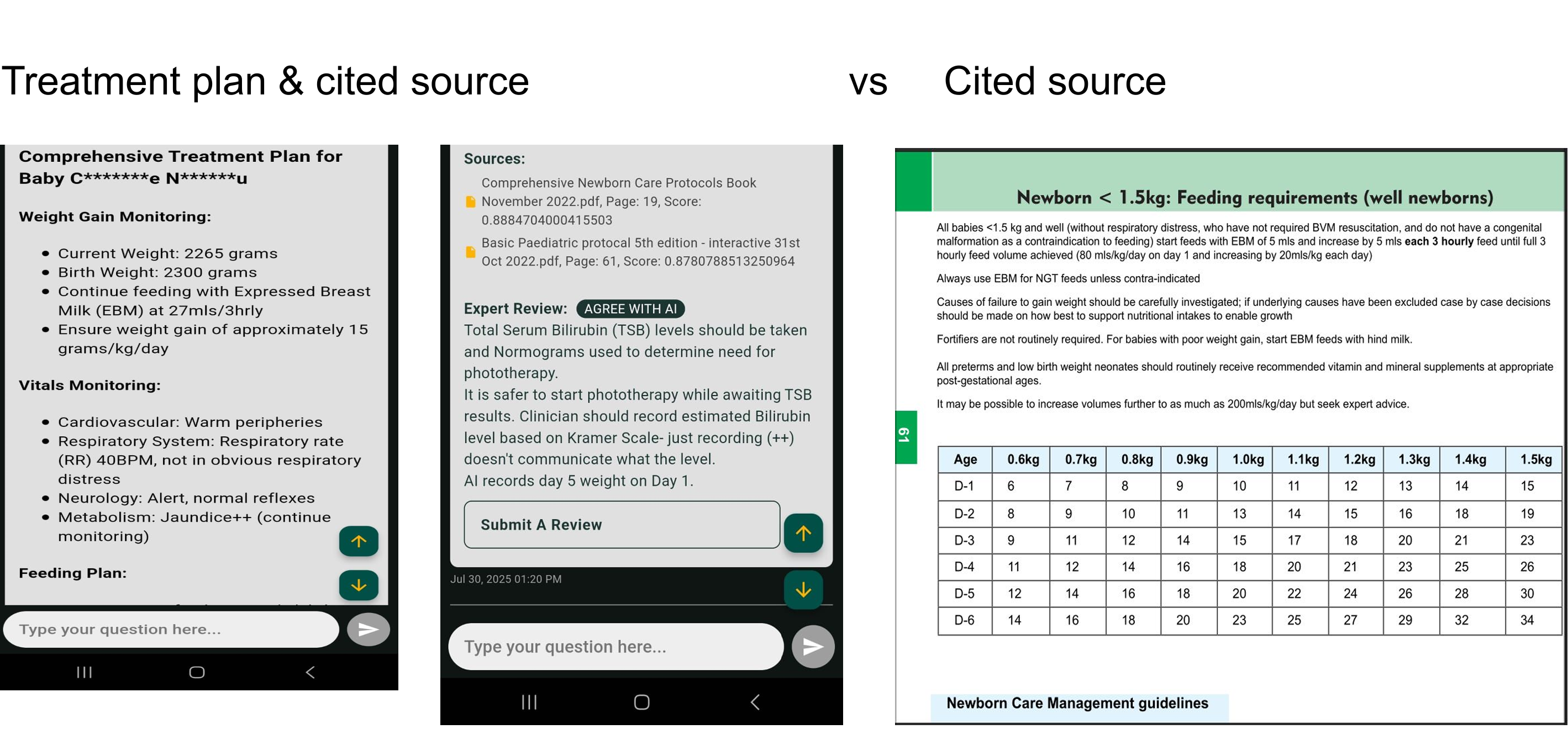


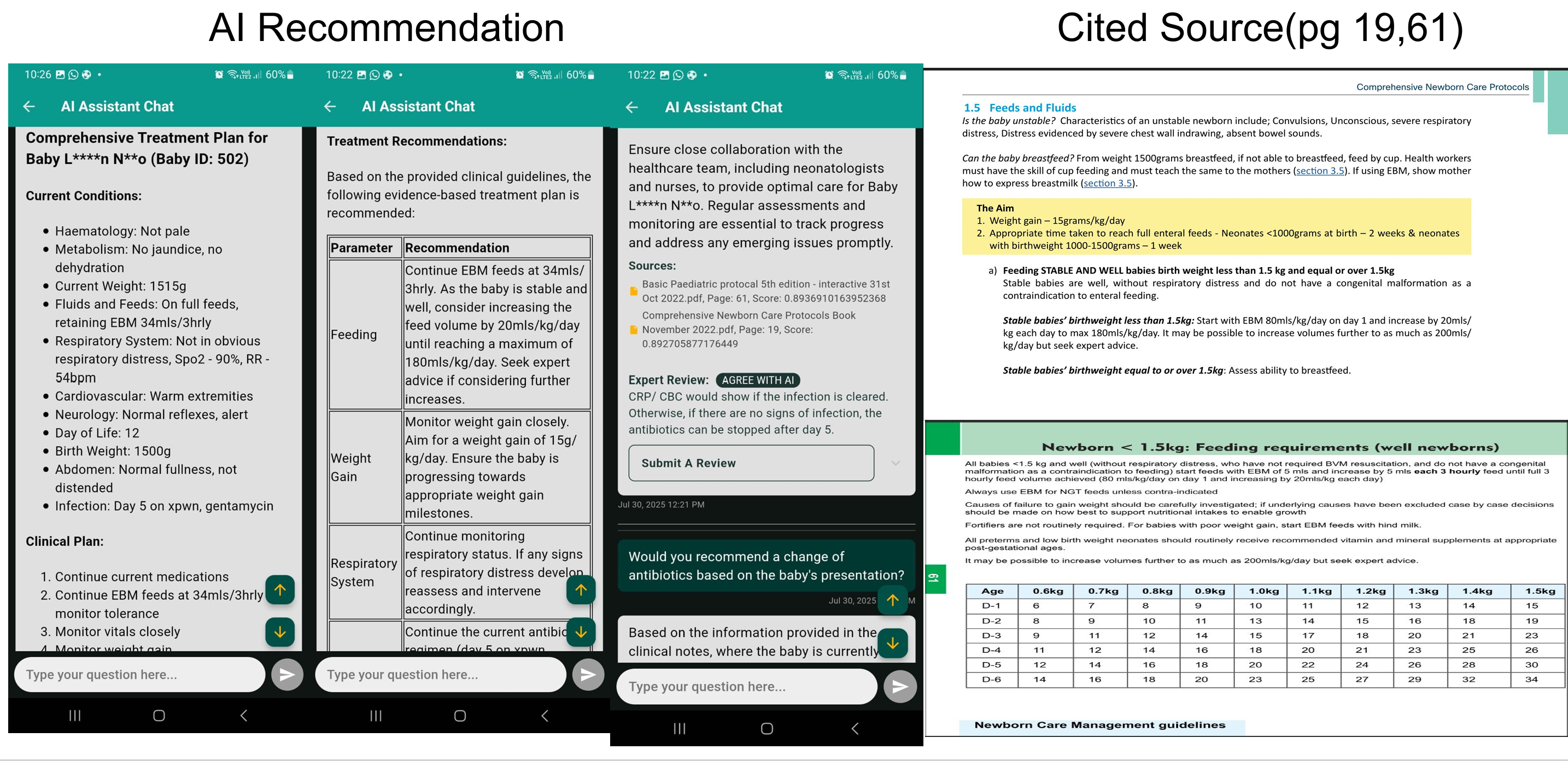


**Figure S1:** Agreement Metrics for AI vs Guideline Evaluation. The figures shows the AI-Generated Recommendations (assessment, diagnosis, and management), and the Citation Data (National guideline document and page number).

| Theme | % | Example Comment |
| --- | --- | --- |
| Dosing / calculation issues | 24.4 | “Day 1 fluids should be 80ml/kg, not 60ml/kg.” |
| Clinical accuracy / interpretation | 19.5 | “AI says bilirubin is below threshold yet recommends intensive phototherapy.” |
| System / database limitations | 17.1 | “Plan looks generic, not customized to this patient.” |
| Missing clinical context | 14.6 | “AI should recommend management of AKI and hypernatremia.” |
| Positive feedback | 14.6 | “AI is right.” |
| Evidence / guidelines | 9.8 | “TSB levels and nomograms should guide phototherapy decisions.” |

**Figure S2:** provides an example of a case that resulted in partial agreement or a disagreeing rating. This figure showcases the discrepancy between the AI output and the National Guidelines, along with the specific Expert Rationale/Comment explaining the systematic error identified.
